# A P7 Phage-Like Plasmid Carrying *mcr-1* in an ST15 *Klebsiella pneumoniae* Clinical Isolate

**DOI:** 10.3389/fmicb.2018.00011

**Published:** 2018-01-22

**Authors:** Weilong Zhou, Lu Liu, Yu Feng, Zhiyong Zong

**Affiliations:** ^1^Center of Infectious Diseases, West China Hospital, Sichuan University, Chengdu, China; ^2^Division of Infectious Diseases, State Key Laboratory of Biotherapy, Chengdu, China; ^3^Department of Infection Control, West China Hospital, Sichuan University, Chengdu, China; ^4^Center for Pathogen Research, West China Hospital, Sichuan University, Chengdu, China

**Keywords:** colistin, resistance, phagemid, plasmids, *Klebsiella pneumoniae*

## Abstract

A *Klebsiella pneumoniae* clinical strain, named SCKP83, was isolated and found to be resistant to colistin thanks to the presence plasmid-borne colistin resistant gene *mcr-1*. The strain was subjected to whole genome sequencing and conjugation experiments. The subsequent analysis indicated that the strain belongs to ST15 and the capsular type K41. In SCKP83, *mcr-1* was carried by a 97.4-kb non-self-transmissible plasmid, a 90.9-kb region of which was predicted as an intact phage. This phage was 47.79% GC content, encoded 105 proteins and contained three tRNAs. *mcr-1* was located downstream of two copies of the insertion sequence IS*Apl1* (one complete and one truncated) and was inserted in the *ant1* gene, which encodes a putative antirepressor for antagonizing C1 repression, in this phage. The phage is highly similar to phage P7 (77% coverage and 98% identity) from *Escherichia coli*. Several similar *mcr-1*-carrying plasmids have been found in *E. coli* at various locations in China, suggesting that these phage-like plasmids have circulated in China. The findings in this study suggest that the P7 phage-like plasmids are not restricted to *E. coli* and may represent new vehicles to mediate the inter-species spread of *mcr-1*.

## Introduction

*Klebsiella pneumoniae* is a major pathogen causing a variety of infections in humans. Colistin is the last resort antimicrobial agent to treat infections caused by *K. pneumoniae* including those with resistance to carbapenems. However, colistin-resistant *K. pneumoniae* have emerged worldwide (Olaitan et al., 2014a). A few mechanisms including both chromosomal and plasmid-borne ones have been identified to be responsible for resistance to colistin in *K. pneumoniae* (Olaitan et al., 2014b). Plasmid-borne colistin resistance genes including *mcr-1* (Liu et al., 2016), *mcr-2* (Xavier et al., 2016), and *mcr-3* (Yin et al., 2017) have been found recently. In particular, *mcr-1* has been identified in various species of the Enterobacteriaceae in many countries (Poirel et al., 2017).

Bacteriophages (phages) are viruses able to infect and replicate within bacteria. Phages mediate the transfer of genetic components between bacteria via transduction. Phages may have a lytic cycle or a lysogenic cycle or both. In the lytic cycle, phage genomes are replicated and are assembled into particles, which cause cell lysis and are then released. In the lysogenic cycle, phage genomes integrate into the chromosome of host bacterial cells to exist in a latent or dormant state without causing cell lysis (Feiner et al., 2015). The structure of phages typically consists of a protein head that encapsulates a DNA or RNA genome and a tail that attacks the bacterial host (Wurtz, 1992). Phage genomes vary remarkbly in form and size but usually encode products for host takeover, replication, virion assembly, or lysis (Black and Thomas, 2012). Some phages may integrate into plasmids and can therefore be transferred by the host plasmid (Oliver et al., 2005; Shin and Ko, 2015).

*mcr-1* is commonly carried by plasmids of the IncI2 or IncX4 replicon type and has also been found on IncF, IncHI2, or IncP plasmids (Poirel et al., 2017). We have found a plasmid carrying *mcr-1* and phage P7-like sequences, which is reported here.

## Methods

### Bacterial strain

*K. pneumoniae* strain SCKP83 was recovered from a sputum sample of a 90-year-old male patient with severe pneumonia in February 2017 in China, who did not receive colistin before. Species identification was performed using Vitek II (bioMérieux, Marcy-l′Étoile, France) and MALTI-TOF (Bruker, Billerica, MA, USA). *In vitro* susceptibility of colistin was performed using the broth dilution method of the Clinical Laboratory Standards Institute (CLSI) (CLSI, 2017) and breakpoints of colistin defined by EUCAST (http://www.eucast.org/) were applied. The presence of plasmid-borne colistin resistant genes *mcr-1, mcr-2*, and *mcr-3* was screened by PCR as described previously (Xavier et al., 2016; Zhao and Zong, 2016; Yin et al., 2017).

### Whole genome sequencing and analysis

The strain was subjected to whole genome sequencing. Genomic DNA was prepared using the QIAamp DNA Mini Kit (Qiagen, Hilden, Germany) and whole genome sequencing was performed using the HiSeq X10 Sequencer (Illumina, San Diego, CA). The coverage was approximately 300 × coverage, which was calculated based on the estimated genome size and the average output of the sequencer. Reads were trimmed using Trimmomatic (version 0.36) (Bolger et al., 2014) and were then assembled to contigs using SPAdes (version 3.11) (Bankevich et al., 2012) with careful mode turned on. Sequence type and capsular type were determined using the genomic sequence to query the multi-locus sequence typing and *wzi* allele databases of *K. pneumoniae* available at http://bigsdb.pasteur.fr/klebsiella/klebsiella.html. Antimicrobial resistance genes were identified from genome sequences using the ABRicate program (https://github.com/tseemann/abricate) and ResFinder (https://cge.cbs.dtu.dk/services/ResFinder/). The plasmid carrying *mcr-1*, designated pMCR_SCKP-LL83, was circularized using PCR and Sanger sequencing to fill in gaps between contigs. Plasmid replicon was determined using the PlasmidFinder tool at http://genomicepidemiology.org/. Similar plasmids were retrieved from the GenBank and pairwise comparisons were preformed using BLASTn alignment (Altschul et al., 1990) and BRIG (Alikhan et al., 2011). The presence of phages was screened using PHASTER (http://phaster.ca/) (Arndt et al., 2016). tRNAs were screened using tRNA-SE (http://lowelab.ucsc.edu/tRNAscan-SE/) (Lowe and Chan, 2016).

### Nucleotide sequence accession numbers

Draft whole-genome sequence of strain SCKP83 has been deposited into GenBank under the accession number NOKM00000000. Short reads of the whole-genome sequence of strain SCKP83 has been deposited into Short Reads Achieve under the accession number SRP099296. The complete sequences of pMCR_SCKP-LL83 has been deposited into GenBank under the accession numbery MF510496.

### Conjugation and transformation experiments

Conjugation experiments were performed using both broth- and filter-based methods as described previously (Coque et al., 2002; Novais et al., 2006; Valenzuela et al., 2007). The azide-resistant *Escherichia coli* strain J53 was used as the recipient and 2 μg/ml colistin plus 150 μg/ml sodium azide were used for selecting transconjugants. Plasmids were prepared from strain SCKP83 using alkaline lysis (Sambrook and Russell, 2001) and were used for electroporation. Electroporation was conducted using a Gene Pulser (Bio-Rad, Hercules, CA, USA) with an electrical pulse of 25 μF capacitance, 2.5 kV and 200 Ω sample resistance. *E. coli* strain DH5α and a colistin-susceptible *K. pneumoniae* strain 020018 were used as recipient strains. Potential transformants were selected on agar plates containing 2 μg/ml colistin.

### Induction of bacteriophage

To determine the nature of pMCR_SCKP-LL83, we performed the induction assay using ultraviolet ray and mitomycin C as described previously (Mitsui et al., 1973; Raya and H'bert, 2009). Briefly, for UV induction, 1 ml culture of strain SCKP-LL83 in the exponential phase was harvested and resuspended in 0.05 M phosphate buffer (pH 6.8). The suspension was adjusted to the 0.5 McFarland turbidity. Six aliquots of 150 μl were spotted on a 9 cm Petri dish and irradiated by a germicidal UV lamp at a distance of 100 cm. The drops were collected at 10, 20, 30, 60, 90, and 120 s serially, each of which was then incubated with 1 ml LB broth under 37°C in dark for 3–4 h. Lysis was observed by naked eyes. For mitomycin C induction, 100 ml cultures of strain SCKP-LL83 were added with mitomycin C to a final concentration of 0.1, 1, 10, 20, and 40 μg/ml and were incubated under 37°C with shaking. Aliquots (1 ml) were sampled at 2, 4, 12, and 24 h. The cultures were filtrated through 0.22 μm polyethersulfone membranes (Merck Millipore, Billerica, MA, USA) and the membranes were used for the plaque formation test, which was carried out via the agar overlay method (Kropinski et al., 2009). All of the tests were performed in triplicate.

### Assay for replication module

The replication initiation protein-encoding gene *repB* and its replication origin sequence (*ori*) of pMCR_SCKP-LL83 were amplified with self-designed primers OriF (CGGAATTCGAAATGGGATCAACATTGACTATACG) and OriR (CGGAATTCATCAATACCACTGCTTGATGAGA; *EcoR*I sites are underlined). The amplicons were cloned onto the vector pKC1139, which has a temperature sensitive origin *oriT* and cannot replicate at temperatures higher than 30°C. The ligated vectors were transformed into *E. coli* DH5α and the transformants were screened by apramycin (100 μg/ml) at 37°C. The presence of *repB* and *ori* in transformants were confirmed by PCR with M13 (-21) Forward and M13-R primers binding to the clone vector and Sanger sequencing.

## Results and discussion

Strain SCKP83 was resistant to colistin (MIC, 8 μg/ml) and had *mcr-1* but no *mcr-2* and *mcr-3* genes. Whole genome sequencing of strain SCKP83 generated 5,247,124 clean reads, which were then assembled to 119 contigs (89 >1,000 bp) with a 50.38% GC content. Strain SCKP83 belonged to ST15, which is a relative common type of *K. pneumoniae* seen in China (Zhang et al., 2017b). The capsular type of strain SCKP83 was K41.

*mcr-1* was carried by a 97.4 kb plasmid, pMCR_SCKP-LL83, which did not carry any additional known antimicrobial resistance genes. Despite repeated attempts, no colistin resistant transconjugants were obtained, suggesting that pMCR_SCKP-LL83 is not self-transmissible. In addition, the transformation of this plasmid into *E. coli* strain DH5α and a colistin-susceptible *K. pneumoniae* strain was unsuccessful. This suggests that this plasmid may be strain-specific or its transformation occurs at a low frequency, which could not be detected in our experiments. pMCR_SCKP-LL83 had a single pO111 plasmid replicon. Transformants containing *repB* and its *ori* were obtained. The presence of *repB* and *ori* allows the temperature sensitive vector pKC1139 to replicate at 37°C, suggesting that the replication module of pMCR_SCKP-LL83 indeed leads to the replication of this plasmid.

**Figure 1:**
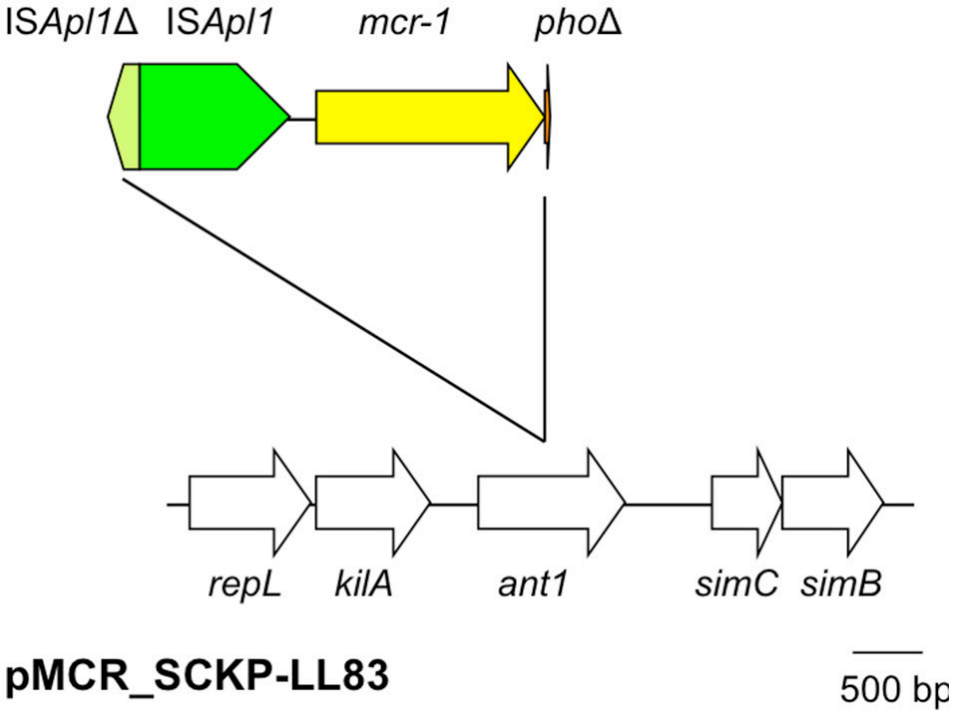
The genetic context of *mcr-1* on pMCR_SCKP-LL83. The IS*Apl1*Δ-IS*Apl1*-*mcr-1*-*pho*Δ structure is inserted into *ant1* but without the 2-bp DR characteristic of the insertion of IS*Apl1*. The two IS*Apl1* are at contrary oppositions. Δ refers to truncated genes or elements. *ant1* encodes a putative antirepressor. The phage genes surrounding *ant1* include *repL* (encoding replication protein), *kliA* (encoding a putative host killing protein), *simB* and *simC* (both encoding proteins for host immunity).

On pMCR_SCKP-LL83, *mcr-1* was located downstream of a complete insertion sequence IS*Apl1*. The phosphoesterase-encoding *pho* gene that is always located downstream of *mcr-1* was truncated at its 3′-end with only 38 bp out of the 747-bp gene remaining. Surprisingly, immediate upstream of the complete IS*Apl1* (1,070 bp in length) lies another IS*Apl1* that is truncated at its 5′-end with the presence of 223 bp including an intact right-hand inverted repeat (IRR) (Figure 1). When we artificially subtract the IS*Apl1*Δ-IS*Apl1*-*mcr-1*-*pho*Δ region from pMCR_SCKP-LL83, the remaining artificially-joining sequence perfectly matched the *ant1* gene, which encodes a putative antirepressor for antagonizing C1 repression by formation of Ant1/Ant2/C1 complex. It therefore becomes evident that the IS*Apl1*Δ-IS*Apl1*-*mcr-1*-*pho*Δ structure is inserted into *ant1*. It has been found that a single copy of IS*Apl1* is able to mobilize *mcr-1* and *pho* together with itself (Li et al., 2017; Zhao et al., 2017). The insertion of IS*Apl1* would generate 2-bp direct target repeats (DR). However, no 2-bp DRs were present flanking the IS*Apl1*Δ-IS*Apl1*-*mcr-1*-*pho*Δ structure, suggesting that the formation of such a complex structure was not directly due to the insertion mediated by IS*Apl1*. The mechanism responsible for generating the IS*Apl1*Δ-IS*Apl1*-*mcr-1*-*pho*Δ structure remains unclear but might have involved recombination.

**Figure 2:**
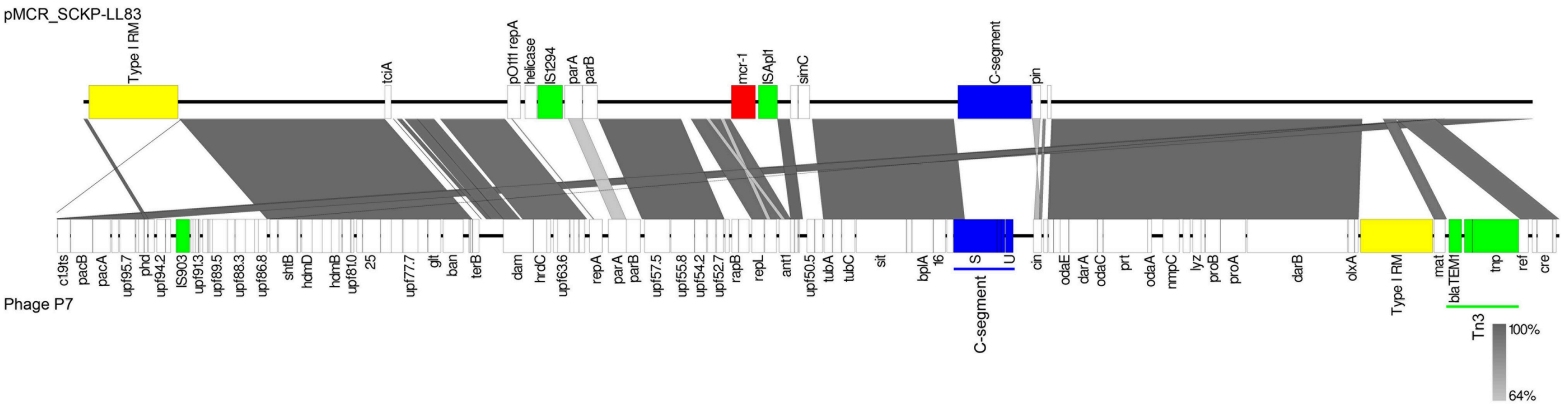
Comparison of pMCR_SCKP-LL83 with phage P7 (GenBank accession no. AF503408). Similar regions are indicated with the degree of nucleotide identity being shown in gray scales. Mobile genetic elements, type I restriction-modification (RM) systems and C-segments are shown in green, yellow, and blue, respectively.

A 90.9-kb region of the 97.4-kb pMCR_SCKP-LL83 was predicted as an intact phage. Neither the appearance of lysis nor the formation of plaques was observed in the UV induction. In mitomycin C induction, no plaques were formed at the tested concentrations and intervals. These results suggest that pMCR_SCKP-LL83 was indeed a plasmid. Nonetheless, the phage region on pMCR_SCKP-LL83 had 47.79% GC content, encoded 105 proteins and contained three tRNAs, i.e., tRNA-Asn, tRNA-Thr, and tRNA-Met (Table 1). pMCR_SCKP-LL83 is highly similar (72% coverage and 98% identity) to the 101.7-kb *Enterobacteria* phage P7 (GenBank accession no. AF503408). Phage P7 (previously called as φ*amp*) was isolated from *E. coli* of human fecal flora (Smith, 1972) and exists as a nonintegrated autonomous circular plasmid that constitutes a unique compatibility group (Hedges et al., 1975). Compared with P7, pMCR_SCKP-LL83 did not have the *bla*_TEM−1_-carrying transposon Tn*3*, the type I restriction-modification system *Eco*P7, a 4-kb invertible C-segment and a few genes, most of which encode proteins of unknown function (Table S1 in the Supplementary file and Figure 2). C-segment contains several genes encoding phage tail fibers and also determines the host specificity of the phage (Iida, 1984). In contrast, pMCR_SCKP-LL83 had a few extra genes including an unnamed type I restriction-modification system, *mcr-1* and a 5-kb putative invertible C-segment (Table S1), which is highly similar (92% coverage and 99% identity) to the multiple DNA inversion region *min* on plasmid p15B of *E. coli* 15T (Sandmeier et al., 1991).

**Table 1:** Features of pMCR_SCKP-LL83.

**Feature^a^**	**Position (start–end)**	**Function**
0001	356–1912	Type I restriction-modification system subunit M
0002	1909–3114	Restriction endonuclease subunit S
0003	3235–6351	Type I restriction enzyme EcoR124II R protein
0004	6616–7122	3′-Phosphatase, 5′-polynucleotide kinase
0005/*pmgS*	7195–8457	Putative morphogenetic protein
0006	8459–8677	Hypothetical protein
0007	8759–9460	Hypothetical protein
0008/*pphA*	9457–10134	Serine/Threonine protein phosphatase
0009/*pmgP*	10131–10757	Putative morphogenetic protein
0010	11259–11414	Hypothetical protein
0011/*pmgM*	11481–12059	Putative morphogenetic function protein
0012	12062–12307	Putative morphogenetic protein
0013	12571–12831	Baseplate protein
0014	12841–14058	Tail protein
0015	14062–14790	Tail protein
0016	14777–15562	Hypothetical protein
0017	15564–16580	Tail length tape measure protein
0018	16573–17205	Putative baseplate protein
0019	17252–18250	Hypothetical protein
0018/*dnaB*	18250–19614	Replicative DNA helicase
0021	19900–19975	tRNA-Met
0024/*tciA*	20250–20675	Putative tellurite or colicin resistance protein
0025	21187–21360	Hypothetical protein
0026	21603–21678	tRNA-Thr
0027	21681–21756	tRNA-Asn
0028/*dmt*	22429–24693	DNA adenine methylase family protein
0029/*rdgC*	24690–25595	Recombination-associated protein RdgC
0030	25588–25872	Hypothetical protein
0031	25857–26096	Hypothetical protein
0032	26335–27123	Hypothetical protein
0033	27163–27585	Outer membrane lytic protein
0034/*upfB*	27763–28155	Hypothetical protein
0035	28048–28311	Hypothetical protein
0036/*repA*	28491–29375	Initiator replication family protein of pO111-like replicon
0037	29668–30477	Helicase
IS*1294*	32106–32205	Insertion sequence
0040/*parA*	32334–33530	Plasmid partition protein A
0041/*parB*	33547–34548	Plasmid partition protein B
0042	34774–36480	Putative baseplate protein
0043	36541–38130	Hypothetical protein
0044	38140–38955	Tail tube protein
0045/*pmgG*	38991–39572	Putative morphogenetic protein
0046/*bplB*	39584–40093	Putative baseplate structural protein
0047	40217–40423	Hypothetical protein
0048	40547–40792	Hypothetical protein
0049/*repL*	40843–41652	Replication protein
0050/*kilA*	41718–42518	Putative host killing protein
0051	42682–43587	Hypothetical protein
0052/*mcr-1*	43541–45166	Colistin resistance
IS*Apl1*	45353–46422	Insertion sequence
IS*Apl1Δ*	46423–46645	Insertion sequence, truncated
0055	46580–46915	Antirepressor protein
0056	46912–47133	Hypothetical protein
0057/*simB*	47561–48031	Superimmunity linked function
0058/*simC*	48039–48818	Superimmunity linked function
*0059*/*pmgC*	49028–49594	putative morphogenetic protein
0060/*tubB*	49605–50216	Major tail tube protein
0061/*pmgB*	50231–51112	Putative morphogenetic protein
0062	51194–54586	Transglycosylase SLT domain protein
0063/*pmgA*	54586–54942	Putative morphogenetic protein
0064	54939–56372	Putative baseplate structural protein
0065	56372–57208	Putative tail tube protein
0066	57287–57721	Putative tail fiber structure or assembly protein
0067	57733–59214	Hypothetical protein
0068	59483–59728	Hypothetical protein
0069	59769–60206	Hypothetical protein
0070	60217–60645	Hypothetical protein
0071	60686–61159	Hypothetical protein
0072	61188–61646	Hypothetical protein
0073/*tfaE*	62160–62771	Prophage tail fiber assembly protein TfaE
0074	62771–63229	Hypothetical protein
0075	63240–63683	Hypothetical protein
0076/*pin*	63773–64345	Site-specific recombinase
0077	64781–65044	Hypothetical protein
0078/*lydA*	65119–65448	Lysis determining protein
0079	65445–65888	Lysis determining protein
0080	65875–66477	Hypothetical protein
0081/*darA*	66479–68398	Hypothetical protein
0082/*ddrA*	68395–68760	Hypothetical protein
0083	68797–71760	Hypothetical protein
0084/*hxr*	71750–72061	Putative repressor protein Hxr
0085/*ompD*	72804–73916	Outer membrane porin protein OmpD
0086/*ssb*	74150–74638	Single-stranded DNA-binding protein
0087/*lys*	74808–75365	Lysozyme
0088	75657–76676	Putative head processing protein
0089	76669–78378	Putative portal protein
0090	78454–85221	Putative DNA adenine methyltransferase
0091	85255–85695	Hypothetical protein
0092	85692–85940	Modulator protein
0093	85982–87286	Hypothetical protein
0094	87343–87984	Maturation control protein
0095/*ref*	88173–88733	Recombination enhancement function protein
0096	88981–89190	Putative lysogeny establishment protein
0097/*cre*	89343–90374	GST-loxP-cre recombinase fusion protein
0098/*cra*	90382–90603	Putative Cre-associated regulatory protein
0099	91208–91417	C1 repressor inactivator
0100	91528–92379	Primary repressor of lytic function
0101	92405–93889	Putative large terminase protein
102/*pacA*	93889–95082	Terminase A protein
0103/*lpa*	95169–95621	Late promoter activating protein
0104	95710–96753	Hypothetical protein
0105	96781–96960	Hypothetical protein
0106/*doc*	96965–97345	Toxin Doc
0001	356–1912	Type I restriction-modification system subunit M
0002	1909–3114	Restriction endonuclease subunit S
0003	3235–6351	Type I restriction enzyme EcoR124II R protein
0004	6616–7122	3′-Phosphatase, 5′-polynucleotide kinase
0005/*pmgS*	7195–8457	Putative morphogenetic protein
0006	8459–8677	Hypothetical protein
0007	8759–9460	Hypothetical protein
0008/*pphA*	9457–10134	Serine/Threonine protein phosphatase
0009/*pmgP*	10131–10757	Putative morphogenetic protein
0010	11259–11414	Hypothetical protein
0011/*pmgM*	11481–12059	Putative morphogenetic function protein
0012	12062–12307	Putative morphogenetic protein
0013	12571–12831	Baseplate protein
0014	12841–14058	Tail protein
0015	14062–14790	Tail protein
0016	14777–15562	Hypothetical protein
0017	15564–16580	Tail length tape measure protein
0018	16573–17205	Putative baseplate protein
0019	17252–18250	Hypothetical protein
0018/*dnaB*	18250–19614	Replicative DNA helicase
0021	19900–19975	tRNA-Met
0024/*tciA*	20250–20675	Putative tellurite or colicin resistance protein
0025	21187–21360	Hypothetical protein
0026	21603–21678	tRNA-Thr
0027	21681–21756	tRNA-Asn
0028/*dmt*	22429–24693	DNA adenine methylase family protein
0029/*rdgC*	24690–25595	Recombination-associated protein RdgC
0030	25588–25872	Hypothetical protein
0031	25857–26096	Hypothetical protein
0032	26335–27123	Hypothetical protein
0033	27163–27585	Outer membrane lytic protein
0034/*upfB*	27763–28155	Hypothetical protein
0035	28048–28311	Hypothetical protein
0036/*repA*	28491–29375	Initiator replication family protein of pO111-like replicon
0037	29668–30477	Helicase
IS*1294*	32106–32205	Insertion sequence
0040/*parA*	32334–33530	Plasmid partition protein A
0041/*parB*	33547–34548	Plasmid partition protein B
0042	34774–36480	Putative baseplate protein
0043	36541–38130	Hypothetical protein
0044	38140–38955	Tail tube protein
0045/*pmgG*	38991–39572	Putative morphogenetic protein
0046/*bplB*	39584–40093	Putative baseplate structural protein
0047	40217–40423	Hypothetical protein
0048	40547–40792	Hypothetical protein
0049/*repL*	40843–41652	Replication protein
0050/*kilA*	41718–42518	Putative host killing protein
0051	42682–43587	Hypothetical protein
0052/*mcr-1*	43541–45166	Colistin resistance
IS*Apl1*	45353–46422	Insertion sequence
IS*Apl1Δ*	46423–46645	Insertion sequence, truncate
0055	46580–46915	Antirepressor protein
0056	46912–47133	Hypothetical protein
0057/*simB*	47561–48031	Superimmunity linked function
0058/*simC*	48039–48818	Superimmunity linked function
*0059*/*pmgC*	49028–49594	Putative morphogenetic protein
0060/*tubB*	49605–50216	Major tail tube protein
0061/*pmgB*	50231–51112	Putative morphogenetic protein
0062	51194–54586	Transglycosylase SLT domain protein
0063/*pmgA*	54586–54942	putative morphogenetic protein
0064	54939–56372	putative baseplate structural protein
0065	56372–57208	Putative tail tube protein
0066	57287–57721	Putative tail fiber structure or assembly protein
0067	57733–59214	Hypothetical protein
0068	59483–59728	Hypothetical protein
0069	59769–60206	Hypothetical protein
0070	60217–60645	Hypothetical protein
0071	60686–61159	Hypothetical protein
0072	61188–61646	Hypothetical protein
0073/*tfaE*	62160–62771	Prophage tail fiber assembly protein TfaE
0074	62771–63229	Hypothetical protein
0075	63240–63683	Hypothetical protein
0076/*pin*	63773–64345	Site-specific recombinase
0077	64781–65044	Hypothetical protein
0078/*lydA*	65119–65448	Lysis determining protein
0079	65445–65888	Lysis determining protein
0080	65875–66477	Hypothetical protein
0081/*darA*	66479–68398	Hypothetical protein
0082/*ddrA*	68395–68760	Hypothetical protein
0083	68797–71760	Hypothetical protein
0084/*hxr*	71750–72061	Putative repressor protein Hxr
0085/*ompD*	72804–73916	Outer membrane porin protein OmpD
0086/*ssb*	74150–74638	Single-stranded DNA-binding protein
0087/*lys*	74808–75365	Lysozyme
0088	75657–76676	Putative head processing protein
0089	76669–78378	Putative portal protein
0090	78454–85221	Putative DNA adenine methyltransferase
0091	85255–85695	Hypothetical protein
0092	85692–85940	Modulator protein
0093	85982–87286	Hypothetical protein
0094	87343–87984	Maturation control protein
0095/*ref*	88173–88733	Recombination enhancement function protein
0096	88981–89190	Putative lysogeny establishment protein
0097/*cre*	89343–90374	GST-loxP-cre recombinase fusion protein
0098/*cra*	90382–90603	Putative Cre-associated regulatory protein
0099	91208–91417	C1 repressor inactivator
0100	91528–92379	Primary repressor of lytic function
0101	92405–93889	Putative large terminase protein
102/*pacA*	93889–95082	Terminase A protein
0103/*lpa*	95169–95621	Late promoter activating protein
0104	95710–96753	Hypothetical protein
0105	96781–96960	Hypothetical protein
0106/*doc*	96965–97345	Toxin Doc

a *Features: genes, mobile genetic elements or C-segments. The allele numbers of genes present on pMCR_SCKP-LL83 are shown*.

It is well known that phages can transfer genetic components between bacterial isolates, but the role of phages in disseminating antimicrobial resistance genes is still a matter of debate (Colavecchio et al., 2017; Enault et al., 2017). Nonetheless, some studies have found that phages are able to transfer genes conferring resistance to aminoglycosides (*aadA, aphA1, strA, strB*), β-lactams (*bla*_CMY−2_, *bla*_CTX−M−9_, *bla*_OXA−2_, *bla*_OXA−20_, *bla*_PSE−1_, *bla*_TEM_), chloramphenicol (*floR*), or tetracycline (*tet*(A), *tet*(B), *tetG, tetO, tetW*) via transduction (Zhang and LeJeune, 2008; Colomer-Lluch et al., 2014; Bearson and Brunelle, 2015; Ross and Topp, 2015; Shousha et al., 2015; Anand et al., 2016). In addition, a recent study has identified that two *E. coli* phages could promote the transformation of plasmids carrying antimicrobial resistance genes (Keen et al., 2017).

**Figure 3:**
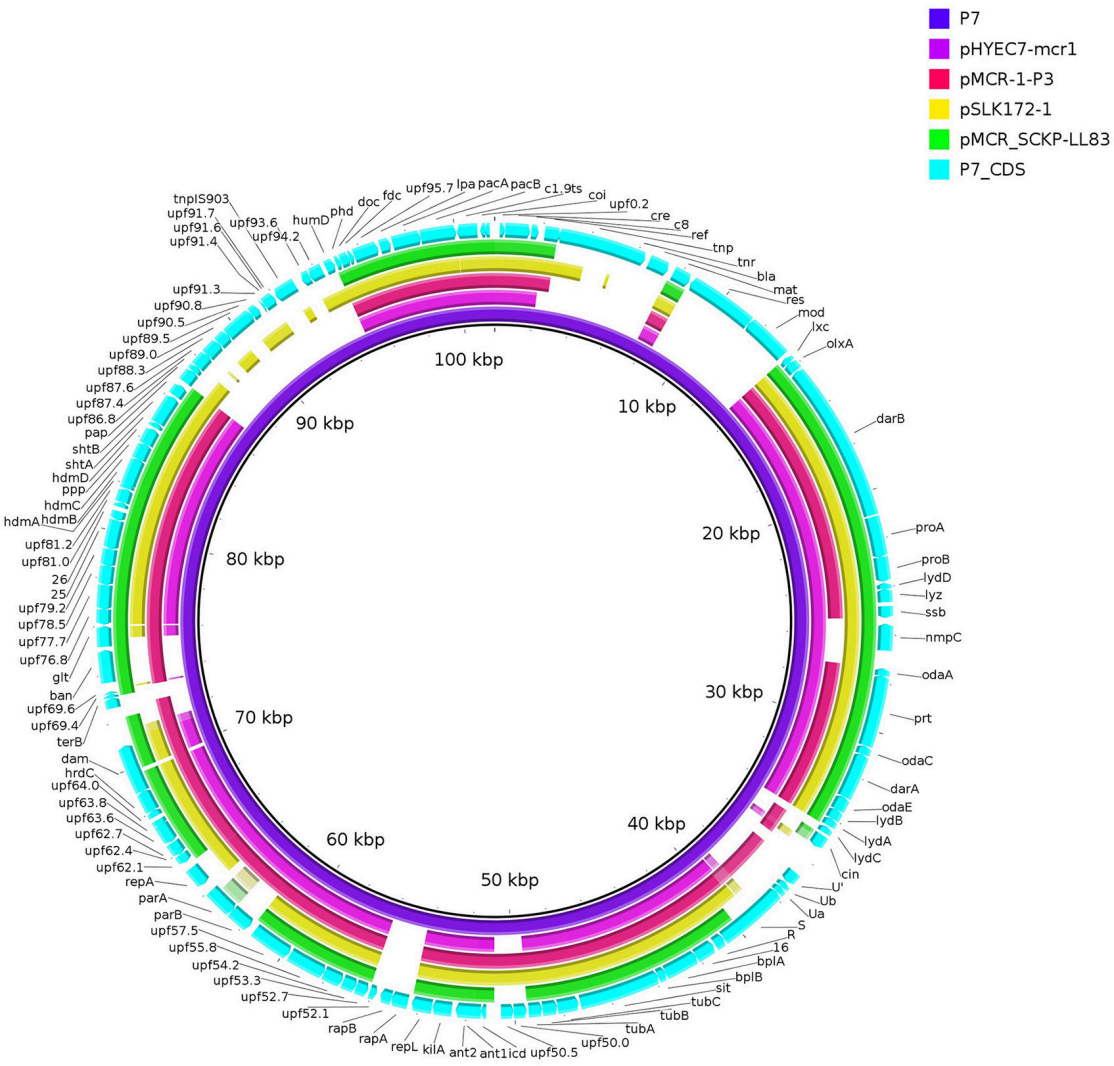
Comparison of phage P7 and similar *mcr-1*-carrying plasmids. The comparison is a pairwise BLASTn alignment performed using BRIG (Alikhan et al., 2011). Plasmids are pMCR_SCKP-LL83 (this study), pHYEC7-mcr1 (GenBank accession no. KX518745) and pSLK172-1 (GenBank accession no. CP017632) and pMCR-1-P3 (GenBank accession no. KX880944). Coding sequences (CDS) of phage P7 (GenBank accession no. AF503408) are indicated. CDS of phage P7 absent from pMCR_SCKP-LL83 or vice-verse are listed in Table S1.

During the process of this work, *mcr-1* in either complete or interrupted version has been found on plasmids containing similar phage sequences including pHYEC7-mcr1 (GenBank accession no. KX518745), pSLK172-1 (GenBank accession no. CP017632) (Bai et al., 2017), and pMCR-1-P3 (GenBank accession no. KX880944) (Zhang et al., 2017a). All of these plasmids have been recovered from *E. coli* at various locations of China and are highly similar (75–79% coverage, 97–99% identity, identified by BLAST; Figure 3) to pMCR_SCKP-LL83. This suggests that the phage sequence-containing plasmids represent new vehicles, which may have circulated in China, to mediate the spread of *mcr-1* in addition to plasmids of IncI2, X4, F, HI2, and P types. The identification of pMCR_SCKP-LL83 from a *K. pneumoniae* is worrisome, suggesting that the P7 phage-like plasmids are not restricted to *E. coli* and may involve in the inter-species spread of *mcr-1*. The various locations of *mcr-1* on these plasmids suggest that these plasmids may have acquired *mcr-1* independently.

In the previous study on the ability of *E. coli* phages to promote the transformation of plasmids carrying antimicrobial resistance gene, phages, and plasmids are separate entities (Keen et al., 2017), which are different from the phage-like plasmid in the present study. As mentioned above, the conjugation and transformation of pMCR_SCKP-LL83 were unsuccessful. Among phage-like plasmids carrying *mcr-1*, pMCR-1-P3 was not self-transmissible and there are no data about whether it can be transferred by transformation (Zhang et al., 2017a), while pSLK172-1 was self-transmissible (Bai et al., 2017). This suggests that some phage-like plasmids may have lost the conjugative module and are therefore not self-transmissible. It is possible that these plasmids acquire genes encoding the conjugative module to become self-transmissible.

In conclusion, we identified and characterized a *mcr-1*-carrying P7 phage-like plasmid from a *K. pneumoniae* clinical isolate. Such phage-like plasmids may represent new types of vehicles to mediate the spread of *mcr-1*.

## Author contributions

ZZ: designed the experiments, analyzed the data, and wrote the MS. LL: performed the experiments and analyzed the data. WZ and YF: analyzed the data.

### Conflict of interest statement

The authors declare that the research was conducted in the absence of any commercial or financial relationships that could be construed as a potential conflict of interest.
