# Supplementary material for: A P7 Phage-Like Plasmid Carrying mcr-1 in an ST15 Klebsiella pneumoniae Clinical Isolate
Source: Front Microbiol. 2018 Jan 22;9:11. doi: 10.3389/fmicb.2018.00011 (PMC5786510; doi:10.3389/fmicb.2018.00011)
Supplement: Supplementary file 1 [file Table1.DOCX]

**Supplementary file**

Table S1. Features absent from pMCR_SCKP-LL83 or from phage P7

| Feature^a^ | Position^b^ (start..end) | Function |  |
| --- | --- | --- | --- |
| Absent from pMCR_SCKP-LL83 | | |  |
| Tn*3* | 2619..7568 | transposon carrying *bla*_TEM-1_ |  |
| *res* | 8500..11412 | Res protein of the restriction-modification system *EcoP*7 |  |
| *mod* | 11415..13355 | modification methylase of the restriction-modification system *EcoP*7 |  |
| *lydC* | 34383..34667 | lysis determinant C |  |
| *cin* | 34789..35349 | site-specific recombinase involved in C-segment inversion |  |
| C-segment | 35353..39361 | invertible DNA element |  |
| upf50.0 | 49507..50046 | membrane protein |  |
| upf50.5 | 50050..50571 | membrane protein |  |
| *rapA* | 54457..55161 | putative replication associated protein |  |
| *rapB* | 55161..55625 | putative replication associated protein |  |
| upf52.1 | 55792..56088 | unknown |  |
| *parA* | 61758..62723 | partition |  |
| *parB* | 62720..63931 | partition |  |
| *terB* | 72606..73040 | tellurite resistance and colicin resistance |  |
| upf86.8 | 86869..87450 | unknown |  |
| upf87.4 | 87443..87703 | unknown |  |
| upf87.6 | 87696..88340 | putative tyrosine protein phosphatase |  |
| upf88.3 | 88328..89014 | unknown |  |
| upf89.0 | 89016..89531 | unknown |  |
| upf89.5 | 89528..90826 | unknown |  |
| upf90.5 | 90519..90713 | unknown |  |
| upf90.8 | 90823..91185 | unknown |  |
| upf91.3 | 91368..91481 | unknown |  |
| upf91.4 | 91482..91790 | unknown |  |
| upf91.6 | 91673..91849 | unknown |  |
| upf91.7 | 91723..91998 | unknown |  |
| IS*903* | 92007..93063 | insertion sequence |  |
| upf93.6 | 93327..93683 | unknown |  |
| upf94.2 | 93684..94268 | unknown |  |
| *humD* | 94443..94832 | DNA repair |  |
|  |  |  |  |
| Absent from P7 | | |  |
| 0001 | 356..1912 | type I restriction-modification system subunit M |  |
| 0002 | 1909..3114 | restriction endonuclease subunit S |  |
| 0003 | 3235..6351 | type I restriction enzyme EcoR124II R protein |  |
| 0024/*tciA* | 20250..20675 | putative tellurite or colicin resistance |  |
| 0036/*repA* | 28491..29375 | initiator replication family protein of pO111-like replicon |  |
| 0037 | 29668..30477 | helicase |  |
| IS*1294* | 30518..32205 | Insertion sequence |  |
| 0040/*parA* | 32334..33530 | partition |  |
| 0041/*parB* | 33547..34548 | partition |  |
| 0047 | 40217..40423 | unknown |  |
| 0048 | 40547..40792 | unknown |  |
| 0052/*mcr-1* | 43541..45166 | colistin resistance |  |
| IS*Apl1* | 45353..46422 | insertion sequence |  |
| IS*Apl1*Δ | 46423..46645 | insertion sequence, truncated |  |
| *simB*/0057 | 47534..48031 | superimmunity linked function |  |
| *simC*0058 | 48039..48818 | superimmunity linked function |  |
| C-segment | 58769..63718 | putative C-segment |  |
| 0076/*pin* | 63773..64345 | site-specific recombinase, DNA-invertase |  |
| 0077/lydC | 64781..65044 | lysis determinant C |  |
| 0093 | 85982..87286 | unknown |  |

^a^Features: genes, mobile genetic elements or C-segments. The allele numbers of genes present on pMCR_SCKP-LL83 are shown.

^b^Positions of features present on P7 but absent from pMCR_SCKP-LL83 refer to the P7 sequence (GenBank accession no. AF503408), while those on pMCR_SCKP-LL83 but absent from P7 refer to the pMCR_SCKP-LL83 sequence
